# A new species of the genus *Opisa* Boeck, 1876 (Crustacea, Amphipoda, Opisidae) and a new record for *Opisatakafuminakanoi* from the East Sea, South Korea

**DOI:** 10.3897/zookeys.1015.60095

**Published:** 2021-02-04

**Authors:** Jun-Haeng Heo, Young-Hyo Kim

**Affiliations:** 1 Department of Life Sciences, Dankook University, 31116, Cheonan, South Korea Dankook University Cheonan South Korea

**Keywords:** Identification key, Lysianassoidea, *Opisaparvimana* sp. nov., parasitic amphipod, taxonomy

## Abstract

Two species of the opisid genus *Opisa* have been collected from the East Sea of South Korea, one of them described as *Opisaparvimana***sp. nov.**. The new species, *O.parvimana***sp. nov.** is similar to *O.odontochela*; however, it can be clearly distinguished from this species because it has 12 blunt robust setae in the palm of gnathopod 1. The other collected species, *Opisatakafuminakanoi* Narahara-Nakano, Kakui & Tomikawa, 2016 is previously known from Japanese waters (southeast of Akkeshi Bay, Hokkaido). Both species are illustrated and compared to related species. A key to *Opisa* species is also provided.

## Introduction

The family Opisidae was first established by Lowry and Stoddart (1995) for a small group of lysianassoid amphipods that currently includes 19 species in four genera (Narahara-Nakano et al. 2016; Horton et al. 2020). They are mainly known from the North Pacific Ocean, North Atlantic Ocean, and Mediterranean Sea (Stoddart and Lowry 2010). Most opisids species are considered ectoparasitic in fish (Vader and Romppainen 1985; Bousfield 1987; Stoddart and Lowry 2010), attracted to the smell of the fish, to which they attach instead of scavenging, because they do not have the mouthpart structure of a scavenger (Stoddart and Lowry 2010). Parasitic amphipods are typically found on slow-moving, slow-growing benthic sharks and bony fishes in cold or deep waters; as a group, rockfish, sculpins, goosefishes, and flatfishes may be described as ambush predators (Vader and Romppainen 1985; Bousfield 1987).

The genus *Opisa* Boeck, 1876 includes a total of four species: *O.eschrichtii* (Krøyer, 1842), *O.odontochela* Bousfield, 1987, *O.tridentata* Hurley, 1963, and *O.takafuminakanoi* Narahara-Nakano, Kakui & Tomikawa, 2016, with the last one reported in Japan (Narahara-Nakano et al. 2016). In this study, we report an additional new species and a newly recorded species of *Opisa* from South Korea through illustrations and text descriptions. This study also provides a key to *Opisa* species around the world.

## Materials and methods

The material examined was collected with a fishing net from subtidal waters of the Namae Port, East Sea, South Korea. Specimens were fixed in 70–80% ethanol and dissected in glycerol on Cobb’s aluminum hole slides. Examinations were performed using a stereoscope (Olympus SZX 10) and a compound microscope (Olympus BX 51), and the drawings and measurements were made with the aid of a drawing tube. The body length was measured from the tip of rostrum to the end of the telson, along the dorsal parabolic line of the body. Nomenclature of the term ‘seta’ follows Watling (1989), Garm and Watling (2013). Terminology of the setae of the mandibular palp follows G. Karaman (1969, 1971) and Lowry and Stoddart (1993). Type specimens are deposited at the National Institute of Biological Resources (**NIBR**), Incheon, South Korea and the Marine Amphipoda Resources Bank of Korea (**MARBK**), Cheonan, South Korea.

## Taxonomy

### Family Opisidae Lowry & Stoddart, 1995

Korean name: Jib-ge-son-gin-pal-yeop-sae-u-gwa, new

#### Genus Opisa

Taxon classificationAnimaliaAmphipodaOpisidae

Boeck, 1876

65C0B03A-0783-5A29-9633-7C4B532D31D7

##### Type species.

*Opisaeschrichtii* Krøyer, 1842

#### Opisa parvimana sp. nov.

Taxon classificationAnimaliaAmphipodaOpisidae

99E6F2BC-DD22-554F-9117-DB2FC86E6C9A

http://zoobank.org/104C5232-D9FA-4CD4-9E9C-110894AF0FAC

##### Type material.

***Holotype***, male, 8.3 mm, MARBK-300 and female, 7.2 mm, MARBK-301, South Korea: Namae Port, Yangyang-gun, Gangwon-do, 37°56'32"N, 128°47'12"E, Y.H. Kim, 21 December 2007. ***Paratypes*** (one male, one female, MARBK-302), same station data as holotype.

##### Diagnosis.

Lateral cephalic lobe subacutely projecting. Mouthparts forming quadrate bundle. Antenna 1, callynophore well developed; flagellum short, 3–5 articles with calceoli in male. Antenna 2, flagellum elongated, with calceoli in male. Upper lip, epistome normal. Mandible, molar setose, left lacinia mobilis blunt. Maxilla 1, outer plate with 11 dentate spine-teeth in an 8/3 crown arrangement. Gnathopod 1, palm straight, armed with a row of 12 blunt robust setae and 1 slender seta, defined by short and subacute process. Uropods 1–2, each ramus with distinct notch with inserted robust setae. Uropod 3, outer ramus biarticulate, longer than inner ramus. Telson cleft.

##### Description.

**Figure 1.**
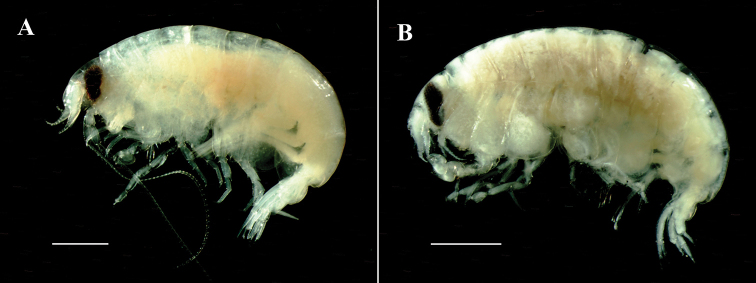
*Opisaparvimana* sp. nov. **A** adult male, MARBK-300, 8.3 mm, habitus **B** adult female, MARBK-301, 7.2 mm, habitus. Scale bars: 1.0 mm (**A, B**).

**Figure 2.**
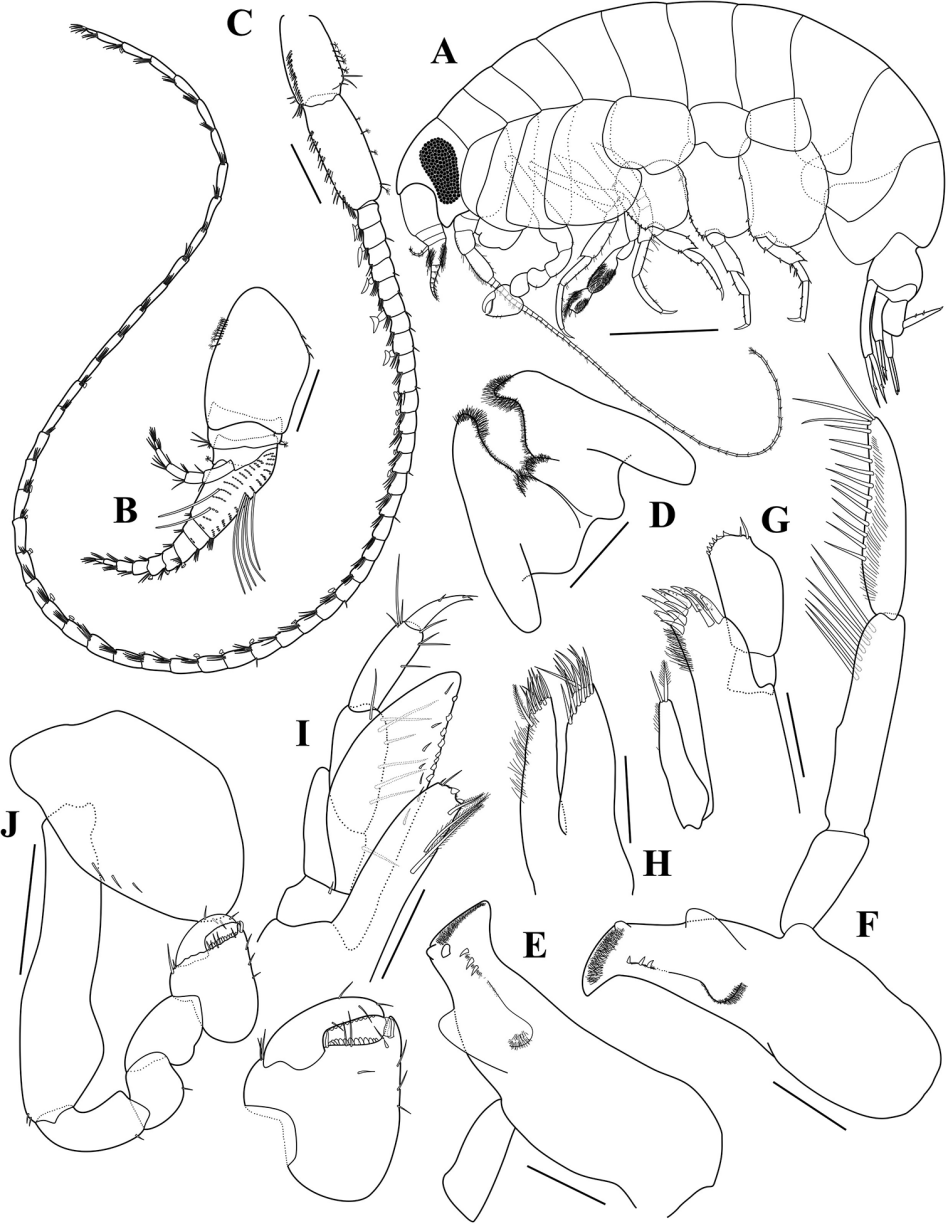
*Opisaparvimana* sp. nov. holotype, adult male, MARBK-300, 8.3 mm **A** habitus **B** antenna 1 **C** antenna 2 **D** lower lip **E** left mandible **F** right mandible **G** maxilla 1 **H** maxilla 2 **I** maxilliped **J** gnathopod 1. Scale bars: 1.0 mm (**A**), 0.2 mm (**B, C**), 0.1 mm (**D–I**), 0.4 mm (**J**).

*Holotype*, *adult male*: body (Figs 1A, 2A) dorsally smooth, 8.3 mm long. Head, lateral cephalic lobe subacute, triangular, slightly concave ventrally; eye large, reniform, black. Epimeron 1 posterior margin smooth and concave; epimeron 2 posteroventral corner right angled; epimeron 3 posteroventral corner rounded. Urosomite 1 with mid-dorsal depression and dorsal carina.

Antenna 1 (Fig. 2B) short, 1.29× head; peduncular article 1 much longer than peduncular articles 2–3 combined, with a row of 10 penicillate setae dorsally; length ratio of peduncular articles 1–3 = 1.00 : 0.28 : 0.17; flagellum 9-articulate, 0.86× shorter than peduncular articles, with 2-field callynophore, calceoli on flagellum articles 3–5; accessory flagellum 5-articulate, article 1 rather elongated.

Antenna 2 (Fig. 2C) slender, elongated, 0.61× body; peduncular article 4 shorter than peduncular article 5, with a row of short setae dorsally, 2 penicillate setae and unequal simple setae anterodistally, 5 penicillate setae ventrally; peduncular article 5 with simple setae dorsally, 4 penicillate setae ventrally; flagellum 55-articulate, calceoli on flagellum articles, some articles missing the calceoli.

Lower lip (Fig. 2D), inner lobes distinct, oval, with pubescence distally; outer lobe with pubescence on distal and medial margins; mandibular lobes elongated.

Left mandible (Fig. 2E), incisor simple, smooth, with a blunt tooth; lacinia mobilis blunt; accessory setal row with 3 robust setae; molar setose, not triturative, as a rounded lobe.

Right mandible (Fig. 2F), incisor smooth, with a blunt tooth; lacinia mobilis absent; accessory setal row with 3 robust setae; palp 3-articulate, attached proximal to molar; article 1 unarmed, short, 0.58× article 3; article 2 longest, with 7 A2-setae; article 3 weakly falcate, 0.88× article 2, with 11 D3-setae and 3 E3-setae.

Maxilla 1 (Fig. 2G), inner plate slender, subrectangular, with 1 pectinate and 1 simple setae apically and setules on outer margin; outer plate with 11 dentate spine-teeth; palp biarticulate, proximal article short, distal article expanded, with 2 slender setae and 6 blunt robust setae apically.

Maxilla 2 (Fig. 2H), inner plate slender, slightly shorter than outer, with 11 apical setae and 1 pectinate seta mediodistally, medial margin with pubescence; outer plate 1.08× longer than inner one, with 13 simple setae distally.

Maxilliped (Fig. 2I), inner plate rectangular, with 3 pectinate setae medially, apical margin with 2 unequal simple setae and 2 blunt robust setae; outer plate moderately expanded, not reaching distal end of article 3 of palp, with 8 blunt robust setae on inner margin and 7 short simple setae medially; palp 4-articulate, article 1 slightly shorter than article 2, with 1 simple seta on inner margin; article 2 with 7 simple setae on inner margin; article 3 slightly shorter than article 2, with simple setae on inner and distal margins; article 4 falcate, 0.47× shorter than article 3.

Gnathopod 1 (Fig. 2J), coxa rounded anterodistally; basis subrectangular, bulge anterodistally; ischium elongated, 0.37 as long as basis, with 1 simple seta posteriorly; carpus unarmed, slightly expanded posteriorly; propodus subequal in length to carpus, ovate, rounded and smooth posteriorly, palm straight, armed with a row of 12 blunt robust setae and 1 slender seta, defined by short and subacute process, with 2 robust setae subapically; dactylus falcate, stout, inner margin evenly dentate.

**Figure 3.**
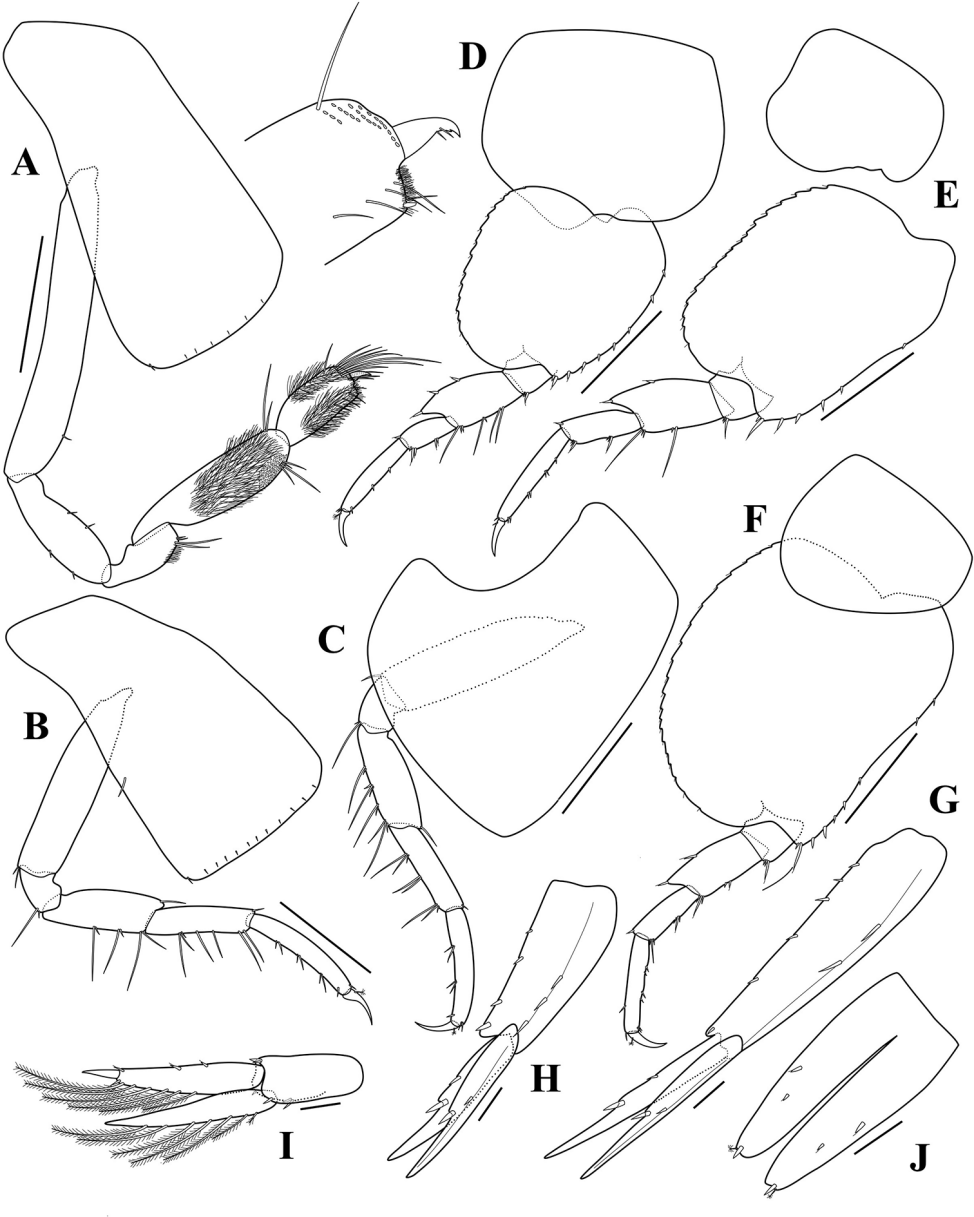
*Opisaparvimana* sp. nov. holotype, adult male, MARBK-300, 8.3 mm **A** gnathopod 2 **B** pereopod 3 **C** pereopod 4 **D** pereopod 5 **E** pereopod 6 **F** pereopod 7 **G** uropod 1 **H** uropod 2 **I** uropod 3 **J** telson. Scale bars: 0.4 mm (**A–G**), 0.1 mm (**H–J**).

Gnathopod 2 (Fig. 3A), coxa subrectangular, slightly widening distally, width 0.49× length; basis slender, elongated, with 1 simple seta anterodistally; ischium elongated, 0.74× carpus, anterior and posterior margins each with 2 simple setae; merus 0.60× ischium, with patch of setules posteriorly and 4 unequal simple setae posterodistally; carpus, posterodistal margin surface with patch of setules, with unequal setae each distal margins, 0.57× basis, posterior margin slightly convex; propodus short, length 2.00× width, subquadrate, surface covered by setules, with cluster of setae anterodistally, palm slightly oblique, with setules, defined by 1 tiny blunt seta posterodistally; dactylus falcate, short, with accessory tooth.

Pereopod 3 (Fig. 3B), coxa similar to that of gnathopod 2, but slightly more widening distally, width 0.49× length; basis slender, with 2 simple setae posterodistally; ischium short, 0.19× basis, with 2 unequal simple setae posterodistally; merus subequal in length to carpus, slightly produced anterodistally, with 1 simple seta anterodistally and 6 unequal simple setae; carpus subrectangular, with 1 simple seta anterodistally, unequal setae posteriorly; propodus subrectangular, slightly shorter than carpus, with long simple setae posteriorly; dactylus falcate, with 1 penicillate seta anteriorly.

Pereopod 4 (Fig. 3C) similar to pereopod 3 except coxa broadened, posterior margin excavate, posterodistal lobe produced, truncate, corner rounded.

Pereopod 5 (Fig. 3D), coxa large, with rounded corners, subquadrate, hind lobe margin angled distally, width subequal to length; basis subcircular, width subequal to length, expanded posteriorly, margin serrate, posteroventral lobe broadly rounded, anterior margin with a row of robust setae; merus expanded posteriorly, anterior margin with 4 simple setae and 3 robust setae, posterior margin with 3 robust setae; carpus 0.56× merus, anterior margin with 2 robust setae and 3 robust setae distally, posterior margin with 1 robust seta distally; propodus rectangular, 1.70× carpus, anterior margin with 3 robust setae; dactylus falcate, with 1 penicillate seta posteriorly.

Pereopod 6 (Fig. 3E), coxa bilobate, anterior lobe small, posterior lobe roundly produced ventrally; basis subquadrate, posterior margin serrate, posteroventral lobe broadly rounded, anterior margin slightly concave, with 7 robust setae; merus expanded posteriorly, anterior margin with 2 long simple and 3 small robust setae, posterior margin with 2 robust setae; carpus 1.36× merus, anterior margin with 2 robust setae and 3 robust setae distally, posterior margin with 1 robust seta distally; propodus rectangular, 1.43× carpus, anterior margin with 3 clusters of 2 robust setae and 1 robust seta distally; dactylus falcate, with 1 penicillate seta posteriorly.

Pereopod 7 (Fig. 3F) similar to pereopod 6, but coxa unilobate; basis much broader than that of pereopod 6, posterior margin broadly expanded.

Uropod 1 (Fig. 3G), peduncle subrectangular, 1.38× outer ramus, with a row of 5 dorsolateral, 2 dorsomedial, and 1 apicolateral robust setae; each ramus with distinct notch with inserted robust setae; outer ramus subequal in length to inner one, both rami each with 1 dorsolateral and 1 dorsomedial robust setae.

Uropod 2 (Fig. 3H), peduncle subequal in length to both rami, with 4 dorsolateral and 3 medial robust setae; each ramus with distinct notch with inserted robust setae; outer ramus subequal in length to inner one, both rami each with 1 dorsolateral and 1 dorsomedial robust setae.

Uropod 3 (Fig. 3I), peduncle short, 0.58× outer ramus, with 2 ventrodistal, 3 dorsomedial, and 1 dorsolateral robust setae; outer ramus biarticulate, 1.06× inner ramus, proximal article with 6 long plumose setae along inner margin and 2 robust setae laterally, each margin with 1 robust seta distally; distal article short, 0.26× proximal one; inner ramus slightly exceed base of distal article of outer ramus, outer margin with a row of 7 plumose setae, inner margin unarmed.

Telson (Fig. 3J) elongated, length 2.05× width, cleft 84% of its length, each lobe with 2 dorsolateral robust setae, 1 robust seta and 1 penicillate seta apically.

**Figure 4.**
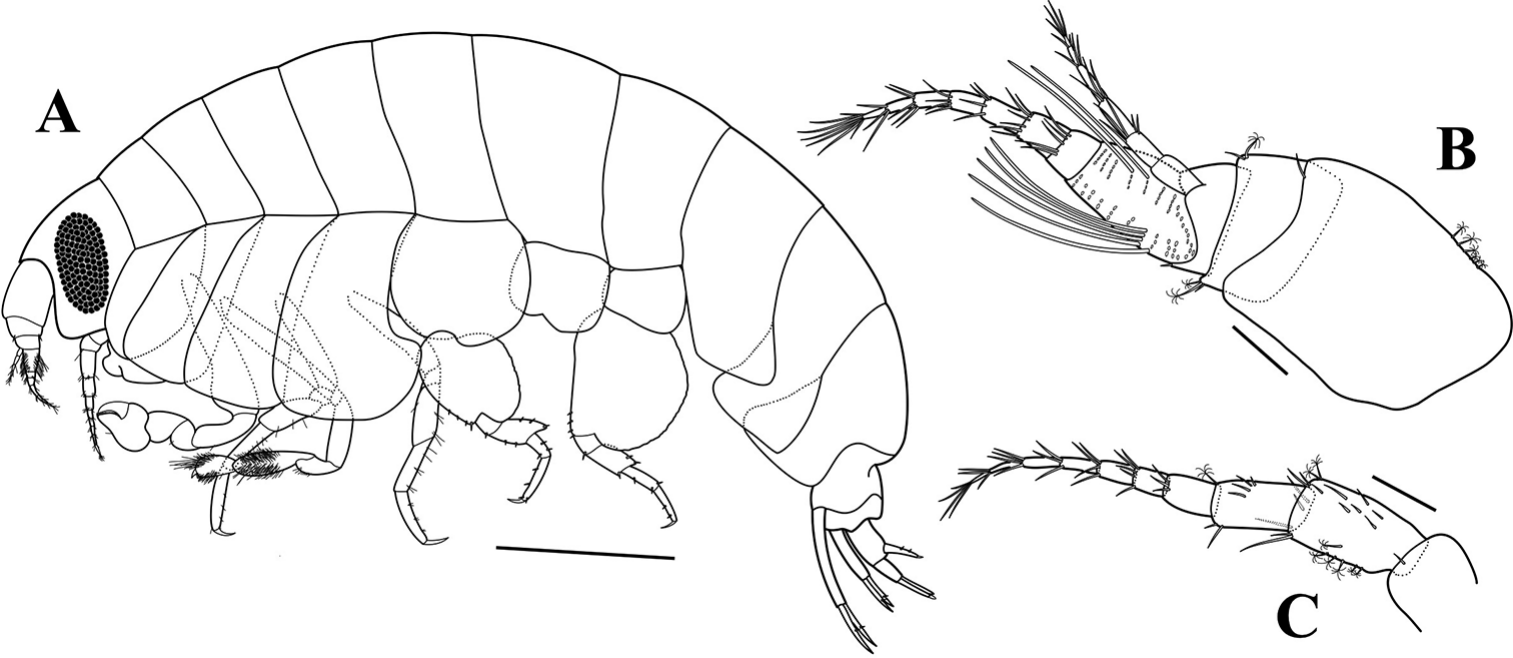
*Opisaparvimana* sp. nov. paratype, adult female, MARBK-301, 7.2 mm **A** habitus **B** antenna 1 **C** antenna 2. Scale bars: 1.0 mm (**A**), 0.1 mm (**B, C**).

*Paratype*, *adult female*: body (Figs 1B, 4A) about 7.2 mm long. Coxa 1 less anteriorly expanded than that of male.

Antenna 1 (Fig. 4B) stout, similar to that of male except peduncular article 1 with 6 penicillate setae dorsally; flagellum 8-articulate, calceoli absent; accessory flagellum 6-articulate, article 1 not elongated.

Antenna 2 (Fig. 4C) slender, much shorter than that of male, peduncular articles 3–5 shorter than those of male; flagellum 7-articulate, calceoli absent.

##### Etymology.

The species name is derived from the Latin *parvus* (=small) and *manus* (=hand) with reference to the relatively small propodus of the gnathopod 1.

##### Remarks.

The genus *Opisa* Boeck, 1876 is similar to the genera *Cheirimedon* Stebbing, 1888, *Normanion* Bonnier, 1893, *Podoprionella* G.O. Sars, 1895, and *Podoprionides* Walker, 1906 in having deep coxal plates, a bilobate telson, small modification or reduction of mandible and maxilliped palps, and distinctly biarticulate outer ramus of uropod 3. However, the genus *Opisa* is easily distinguished from these genera by the following features: 1) enlarged gnathopod 1, strongly subchelate or cheliform; 2) mandibular molar very reduced or even missing; and 3) maxilliped, broadened outer plate and reduced palp (Bousfield 1987).

*Opisaparvimana* sp. nov. is similar to *O.odontochela* Bousfield, 1987 based on the following characteristics: 1) gnathopod 1 with upwardly directed dactylus; 2) gnathopod 1, palm with lined robust setae; 3) gnathopod 2 with single palmar robust seta; and 4) uropods 1 and 2, rami with a robust seta on mid-dorsal margin. However, the new species differs from *O.odontochela* in the following characteristics (compared with the characteristics of *O.odontochela* in parentheses): 1) gnathopod 1, palm with 12 blunt robust setae (vs. about 24-toothed rods); 2) uropod 3, margins of rami with robust setae and plumose setae (vs. margins unarmed).

##### Distribution.

South Korea (East Sea).

#### Opisa takafuminakanoi

Taxon classificationAnimaliaAmphipodaOpisidae

Narahara-Nakano, Kakui & Tomikawa, 2016

8553D20C-D022-5216-95B8-A2843FDABA0E

Opisa takafuminakanoi Narahara-Nakano, Kakui & Tomikawa, 2016: 335, figs 1,2.

##### Material examined.

Male, 8.8 mm, NIBRIV0000880624 and female, 8.7 mm, NIBRIV0000880625, South Korea: Namae Port, Yangyang-gun, Gangwon-do, 37°56'32"N, 128°47'12"E, Y.H. Kim, 21 December 2007. The remaining specimens (two males, three females), same station data as description specimens.

##### Diagnosis.

Lateral cephalic lobe rounded. Mouthparts forming subquadrate bundle. Antenna 1, callynophore well developed; flagellum short, calceoli absent. Antenna 2, flagellum elongated, calceoli absent. Upper lip, epistome normal. Mandible, molar setose, left lacinia mobilis vestigial. Maxilla 1, outer plate with 11 dentate spine-teeth in an 8/3 crown arrangement. Gnathopod 1 enlarge, palm strongly concave, with unequal simple setae, defined by 2 robust setae subapically. Uropods 1–2, each ramus without notch. Uropod 3, outer ramus biarticulate, longer than inner ramus. Telson cleft.

##### Description.

**Figure 5.**
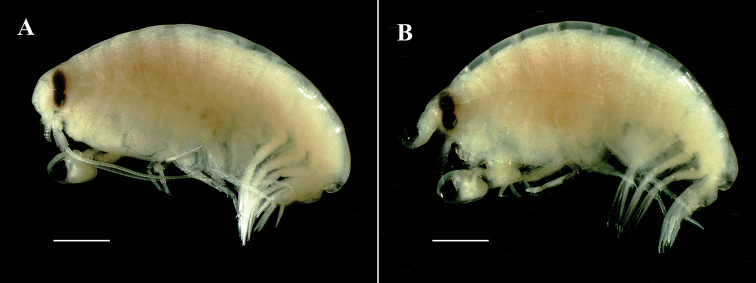
*Opisatakafuminakanoi* Narahara-Nakano, Kakui & Tomikawa, 2016 **A** adult male, NIBRIV0000880624, 8.8 mm, habitus **B** adult female, NIBRIV0000880625, 8.7 mm, habitus. Scale bars: 1.0 mm (**A, B**).

**Figure 6.**
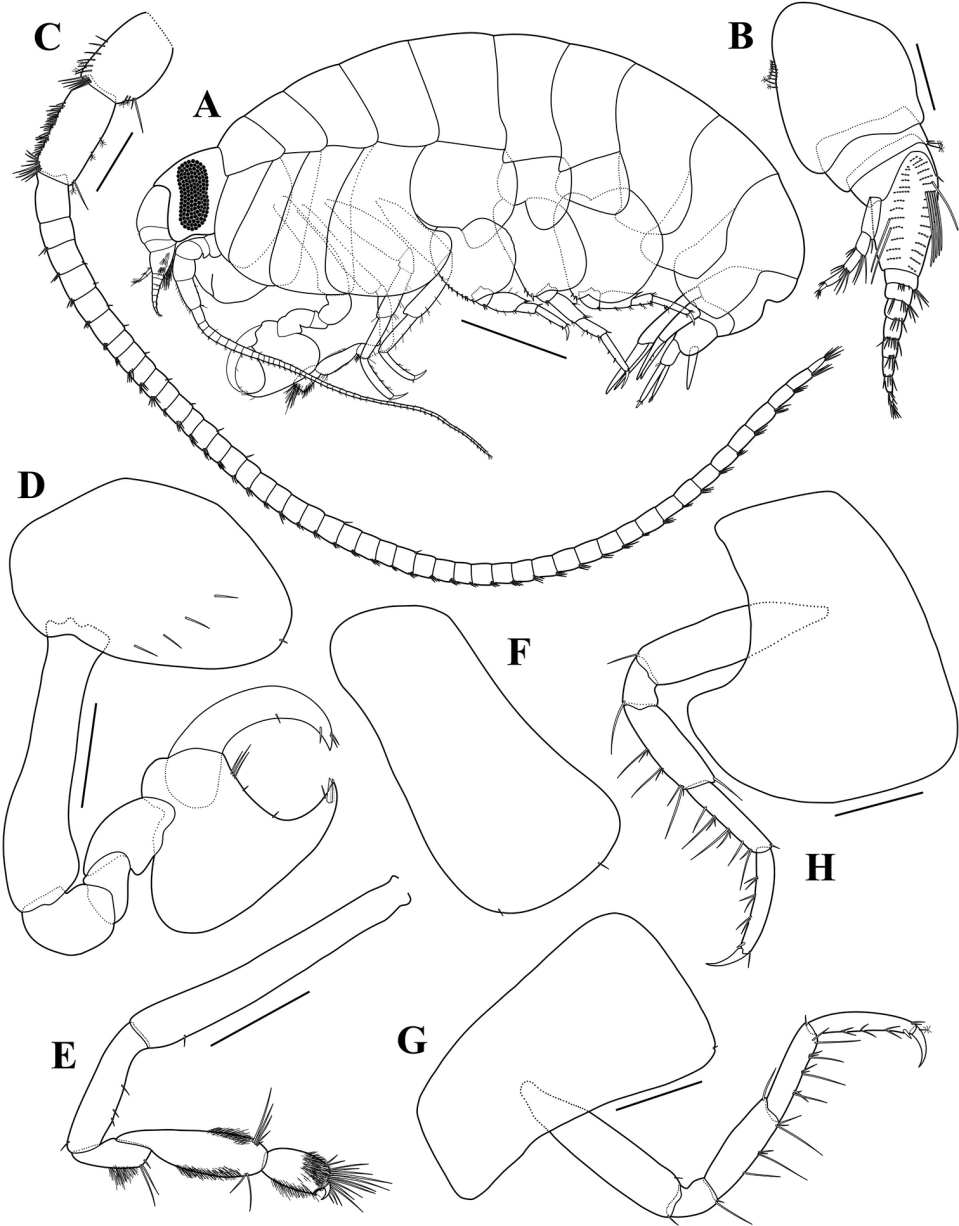
*Opisatakafuminakanoi* Narahara-Nakano, Kakui & Tomikawa, 2016, adult male, NIBRIV0000880624, 8.8 mm **A** habitus **B** antenna 1 **C** antenna 2 **D** gnathopod 1 **E** gnathopod 2 **F** coxa 2 **G** pereopod 3 **H** pereopod 4. Scale bars: 1.0 mm (**A**), 0.2 mm (**B, C**), 0.4 mm (**D–H**).

*Adult male*: body (Figs 5A, 6A) 8.8 mm long, dorsally smooth. Lateral cephalic lobe rounded. Eye large, reniform, black. Epimeron 1 with rounded-quadrate posteroventral corner; epimeron 2 posteroventral corner right angled; epimeron 3 subquadrate. Urosomite 1 with mid-dorsal depression and dorsal carina.

Antenna 1 (Fig. 6B) short, 1.71× head; peduncular article 1 much longer than peduncular articles 2–3 combined, with a row of 9 penicillate setae dorsally; length ratio of peduncular articles 1–3 = 1.00 : 0.31 : 0.25; flagellum 10-articulate, 0.86× shorter than peduncular articles, with 2-field callynophore, calceoli absent; accessory flagellum 5-articulate, article 1 slightly elongated.

Antenna 2 (Fig. 6C) slender and elongated; peduncular article 4 shorter than peduncular article 5, with a row of small setae dorsally, 2 penicillate setae dorsodistally, 6 simple setae distally, 2 penicillate setae and 2 unequal simple setae ventrodistally; peduncular article 5 rectangular, with a row of simple setae dorsally and a cluster of long simple setae, 2 penicillate setae ventrally, 1 long simple and 1 penicillate setae ventrodistally; flagellum 48-articulate, calceoli absent.

Gnathopod 1 (Fig. 6D) strongly chelate, enlarge; coxa rounded anterodistally; basis subrectangular, slightly bulge distally; ischium 0.32 as long as basis, unarmed; carpus 1.25× ischium; propodus enlarge, strong, developed posteriorly, palm strongly concave, with unequal simple setae on palmar margin, defined by 2 robust setae subapically, 1.80× carpus; dactylus stout, strongly curved.

Gnathopod 2 (Fig. 6E), coxa (Fig. 6F) subrectangular, slightly widening distally, width 0.51× length; basis slender, elongated, with 1 simple anterodistal seta; ischium elongated, slightly shorter than carpus, with 3 anterior and 1 posterodistal setae; merus 0.58× ischium, with patch of setules posteriorly and 3 unequal simple setae posterodistally; carpus elongated, 0.49× basis, anterior and posterior margins covered with setules and with distal unequal group of setae, posterior margin slightly convex; propodus short, length 1.86× width, subquadrate, surface covered by setules, with cluster of setae anterodistally, palm oblique, defined by 2 tiny blunt robust setae posterodistally; dactylus falcate, short.

Pereopod 3 (Fig. 6G), coxa similar to that of gnathopod 2, but slightly more widening distally, width 0.60× length; basis slender, with 2 simple setae distally; ischium short, 0.25× basis, with 2 unequal simple setae posterodistally; merus subequal in length to carpus, slightly produced anterodistally, with 1 simple anterodistal seta and 7 unequal simple setae posteriorly; carpus subrectangular, with 1 simple anterodistal seta, 3 clusters of unequal posterior setae, and 2 simple setae posterodistally; propodus subrectangular, subequal in length to carpus, with a paired setae on posterior margin and 1 robust seta posterodistally; dactylus falcate, with 1 penicillate seta anteriorly.

Pereopod 4 (Fig. 6H) similar to pereopod 3 except coxa broadened, posterior margin excavate, posterodistal lobe produced, truncate, corner rounded.

**Figure 7.**
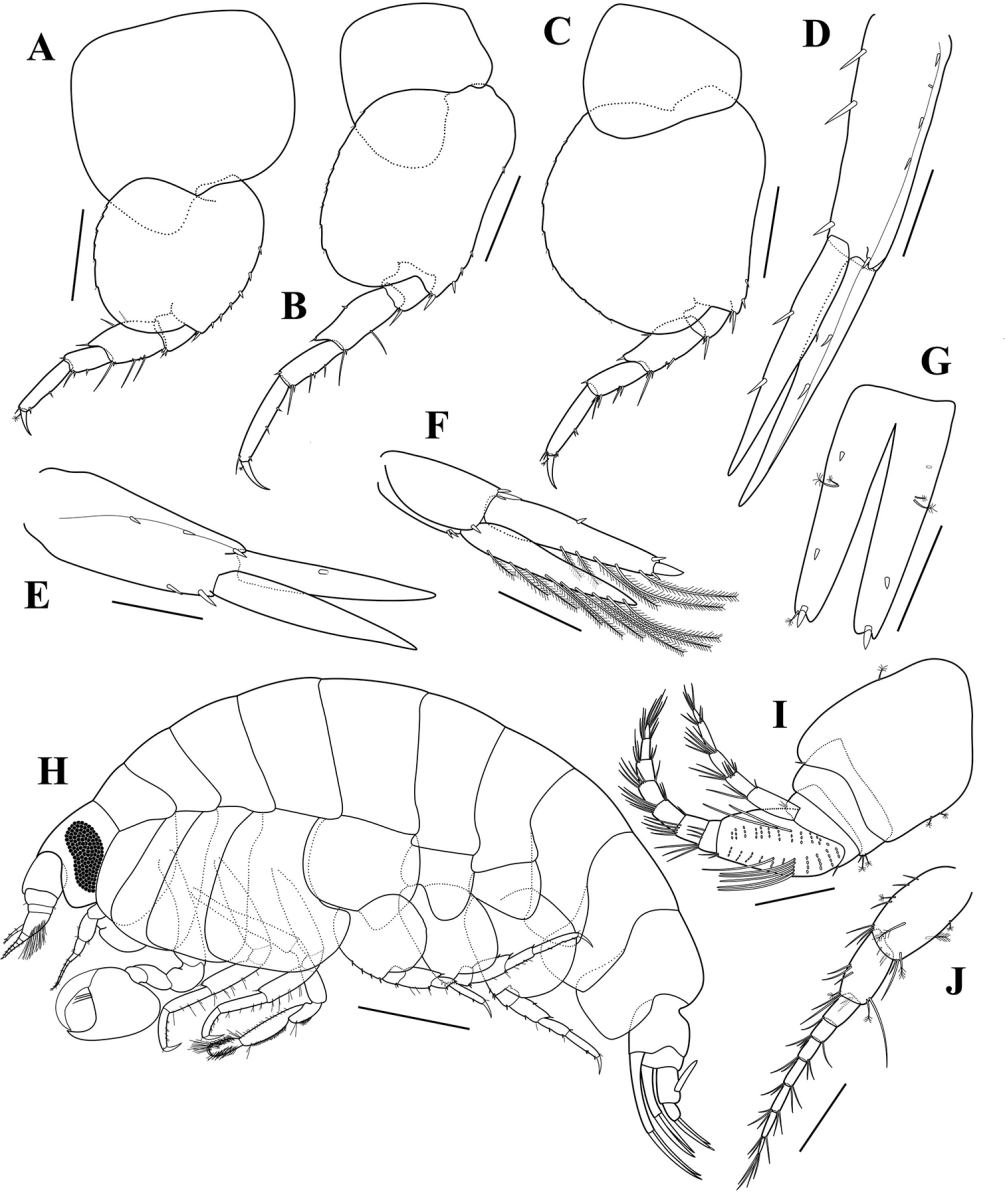
*Opisatakafuminakanoi* Narahara-Nakano, Kakui & Tomikawa, 2016, adult male, NIBRIV0000880624, 8.8 mm **A** pereopod 5 **B** pereopod 6 **C** pereopod 7 **D** uropod 1 **E** uropod 2 **F** uropod 3 **G** telson. Adult female, NIBRIV0000880625, 8.7 mm **H** habitus **I** antenna 1 **J** antenna 2. Scale bars: 0.4 mm (**A–C**), 0.2 mm (**D–G, I–J**), 1.0 mm (**H**).

Pereopod 5 (Fig. 7A), coxa large, with rounded corners, bilobate, posteroventral lobe developed, width 1.24× length; basis subcircular, length 0.90× width, posteriorly expanded, margin serrate, posteroventral lobe broadly rounded, anterior margin with a row of robust setae; merus expanded posteriorly, anterior margin with 3 simple setae and 4 robust setae, posterior margin with 3 simple setae; carpus 0.62× merus, anterior margin with 2 robust setae and 1 simple seta distally, posterior margin with 1 robust seta distally; propodus rectangular, 1.50× carpus, anterior margin with 2 robust setae; dactylus falcate, with 1 penicillate seta posteriorly.

Pereopod 6 (Fig. 7B), coxa bilobate, anterior lobe small, posterior lobe strongly protruding downward; basis subquadrate, posterior margin weakly serrate, posteroventral lobe broadly rounded, anterior margin slightly concave, with 6 robust setae; merus slightly expanded posteriorly, anterior margin with 2 long simple setae and 1 small robust seta, posterior margin with 2 robust setae; carpus 0.75× merus, anterior margin with 3 simple setae and 3 robust setae, posterior margin with 1 robust seta distally; propodus rectangular, 1.42× carpus, anterior margin with 2 robust setae and 1 simple seta distally; dactylus falcate, with 1 penicillate seta posteriorly.

Pereopod 7 (Fig. 7C) similar to pereopod 6, but coxa unilobate; basis much broader than that of pereopod 6, posterior margin broadly expanded.

Uropod 1 (Fig. 7D), peduncle subrectangular, 0.86× outer ramus, with a row of dorsolateral robust setae, 3 dorsomedial and 1 apicolateral robust setae; inner ramus with 2 lateral and 1 medial robust setae, outer ramus slightly longer than inner one, with 2 lateral robust setae.

Uropod 2 (Fig. 7E), peduncle slightly longer than outer ramus, with 3 dorsolateral and 2 dorsomedial robust setae; inner ramus unarmed, unconstricted, subequal in length to outer one; outer ramus with 1 lateral robust seta.

Uropod 3 (Fig. 7F), peduncle short, 0.61× outer ramus, with 2 dorsomedial, 1 dorsolateral, and 3 ventrodistal robust setae; outer ramus biarticulate, 1.09× inner ramus, proximal article with 5 plumose setae along inner margin and 1 lateral robust seta, each margin with 1 robust seta distally; distal article short, 0.19× proximal one; inner ramus nearly reach base of distal article of outer ramus, outer margin with 9 plumose setae, inner margin unarmed.

Telson (Fig. 7G) elongated, length 2.25× width, cleft 87% of its length, dorsolaterally each lobe with 2 small robust setae and 2 unequal penicillate setae, apically with 1 stout seta and 1 penicillate seta.

*Adult female*: body (Figs 5B, 7H) about 8.7 mm long. Head similar to that of male except more rounded lateral cephalic lobe.

Antenna 1 (Fig. 7I) stout, similar to that of male except peduncular article 1 with 1 penicillate seta dorsally and 2 penicillate setae ventrally; flagellum 8-articulate, calceoli absent; accessory flagellum 5-articulate, article 1 slightly elongated.

Antenna 2 (Fig. 7J) slender, much shorter than that of male, peduncular articles 4–5 shorter than those of male; flagellum 7-articulate, calceoli absent.

##### Remarks.

*Opisatakafuminakanoi* Narahara-Nakano, Kakui & Tomikawa, 2016 is similar to *O.eschrichtii* (Krøyer, 1842) in terms of the following characteristics: 1) epimeron 3 round and smooth posteriorly; 2) gnathopod 1 enlarged, with strongly arched dactylus; 3) gnathopod 1, without “palisade” palmar robust setae; 4) coxa 5 longer than length of basis; and 5) uropod 3, rami with plumose setae. However, *O.takafuminakanoi* is distinguished from *O.eschrichtii* by a vestigial lacinia mobilis on the left mandible, the developed posterior lobe of coxa 5, and the unarmed inner ramus of uropod 2. Our specimens are consistent with the original description provided by Narahara-Nakano et al. (2016).

##### Distribution.

Japan, South Korea (East Sea).

#### Key to the species of genus *Opisa*

Modified from Narahara-Nakano et al. 2016.

**Table:** 

1	Epimeron 3, posterior margin smooth; maxilliped, outer plate not reaching distal margin of palp article 3	**2**
–	Epimeron 3, posterior margin crenulated or denticulated; maxilliped, outer plate almost reaching distal margin of palp article 3	***O.tridentata***
2	Gnathopod 1, chela small, dactylus nearly straight, palm of propodus straight, lined with a row of robust setae	**3**
–	Gnathopod 1, chela large, dactylus strongly curved, palm of propodus concave, without a row of robust setae	**4**
3	Gnathopod 1, palm of propodus lined with close-set “palisade” robust setae; uropod 3, rami without marginal setae	***O.odontochela***
–	Gnathopod 1, palm of propodus lined with blunt robust setae; uropod 3, rami with marginal setae	***O.parvimana* sp. nov.**
4	Left mandible, lacinia mobilis developed; coxa 5, posterior lobe weakly developed; uropod 2, inner ramus with robust setae	***O.eschrichtii***
–	Left mandible, lacinia mobilis vestigial; coxa 5, posterior lobe well developed; uropod 2, inner ramus without robust setae	***O.takafuminakanoi***

## Supplementary Material

XML Treatment for Opisa

XML Treatment for Opisa parvimana

XML Treatment for Opisa takafuminakanoi

## References

[B1] Bousfield EL (1987) Amphipod parasites of fishes of Canada. Canadian Bulletin of Fisheries and Aquatic Sciences 217: 1–37.

[B2] Boeck A (1876) De Skandinaviske og Artiske Amphipoder. A.W. Brögger, Christiania, 190–192.

[B3] Bonnier J (1893) Les amphipodes du Boulonnais (1). Bulletin scientifique de la France et de la Belgique 24: 161–207. 10.5962/bhl.part.7782

[B4] Garm A Watling L (2013) The crustacean integument: setae, setules, and other ornamentation. The Natural History of Crustacea, 1, Functional Morphology and Diversity, 167–198. 10.1093/acprof:osobl/9780195398038.003.0006

[B5] Horton T Lowry J De Broyer C Bellan-Santini D Coleman CO Corbari L Costello MJ Daneliya M Dauvin JC Fišer C Gasca R Grabowski M Guerra-García JM Hendrycks E Hughes L Jaume D Jazdzewski K Kim YH King R Krapp-Schickel T LeCroy S Lörz AN Mamos T Senna AR Serejo C Sket B Souza-Filho JF Tandberg AH Thomas JD Thurston M Vader W Väinölä R Vonk R White K Zeidler W (2020) World Amphipoda Database. World Register of Marine Species. http://www.marinespecies.org

[B6] Hurley DE (1963) Amphipoda of the family Lysianassidae from the west coast of North and Central America. Allan Hancock Foundation Occasional Papers 25: 1–160.

[B7] Karaman GS (1969) XXVII. Beitrag zur kenntnis der Amphipoden. Arten der genera *Echinogammarus* Stebb. und *Chaetogammarus* Mart. an der jugoslawischer adriaküste. Glasnik Republičkog zavoda za zaštitu prirode i Prirodnjačke zbirke u Titogradu 2: 59–84.

[B8] Karaman GS (1971) XIX. Beitrag Zur Kenntnis Der Amphipoden. Eine Neue Art Der Gattung *Sarothrogammarus* (Gammaridae) Aus Afghanistan, *S.ruffoi* n. sp. Crustaceana 20(2): 199–207. 10.1163/156854069X00222

[B9] Krøyer H (1842) Une nordiske Slægter og Arter af Amfipodernes Orden, henhørende til Familien Gammarina. (Foreløbigt Uddrag af et større Arbejde). Naturhistorisk Tidsskrift 4: 141–166.

[B10] Lowry JK Stoddart HE (1993) CrustaceaAmphipoda: Lysianassoids from Philippine and Indonesian waters. Résultats des campagnes MUSORSTOM 10(156): 55–109.

[B11] Lowry JK Stoddart HE (1995) The Amphipoda (Crustacea) of Madang Lagoon: Lysianassidae, Opisidae, Uristidae, Wandinidae and Stegocephalidae. Records of the Australian Museum Supplement 22: 97–174. 10.3853/j.0812-7387.22.1995.122

[B12] Narahara-Nakano Y Kakui K Tomikawa KO (2016) *Opisatakafuminakanoi*, a new species of Opisidae from Hokkaido, Japan (Crustacea: Amphipoda). Zootaxa 4200(2): 335–339. 10.11646/zootaxa.4200.2.9 27988625

[B13] Sars GO (1895) Amphipoda. An account of the Crustacea of Norway with short descriptions and figures of all the species 1: 1–240. https://www.biodiversitylibrary.org/page/1256741

[B14] Stebbing TRR (1888) Report on the Amphipoda collected by HMS Challenger during the years 1873–1876. Report on the Scientific Results of the Voyage of HMS Challenger during the Years 1873–1876, Zoology 29: 638–642.

[B15] Stoddart HE Lowry JK (2010) The family Opisidae (Crustacea: Amphipoda: Lysianassoidea) in Australasian waters. Zootaxa 2479(1): 22–38. 10.11646/zootaxa.2479.1.2

[B16] Vader W Romppainen K (1985) Notes on Norwegian marine Amphipoda. 10. Scavengers and fish associates. Fauna norvegica series A 6: 3–8.

[B17] Walker AO (1906) Preliminary descriptions of new species of Amphipoda from the ‘Discovery’ Antarctic Expedition, 1902–1904. Annals and Magazine of Natural History, Series 7 17: 452–458. 10.1080/00222930608562555

[B18] Watling L (1989) A classification system for crustacean setae based on the homology concept. In: Felgenhauer B Watling L Thistle AB (Eds) Functional morphology of feeding and grooming in Crustacea.Balkema, Rotterdam, 15–26. 10.1201/9781003079354-2

